# Violence against doctors in Iraq during the time of COVID-19

**DOI:** 10.1371/journal.pone.0254401

**Published:** 2021-08-06

**Authors:** Riyadh Lafta, Noor Qusay, Meighan Mary, Gilbert Burnham

**Affiliations:** 1 Department of Family and Community Medicine, Al Mustansiriya University, Baghdad, Iraq; 2 Department of International Health, The Johns Hopkins Bloomberg School of Public Health, Baltimore, MD, United States of America; China University of Mining and Technology, CHINA

## Abstract

**Objectives:**

This study assessed patterns in reported violence against doctors working in 11 Baghdad hospitals providing care for patients with COVID-19 and explored characteristics of hospital violence and its impact on health workers.

**Methods:**

Questionnaires were completed by 505 hospital doctors (38.6% male, 64.4% female) working in 11 Baghdad hospitals. No personal or identifying information was obtained.

**Findings:**

Of 505 doctors, 446 (87.3%) had experienced hospital violence in the previous 6 months. Doctors reported that patients were responsible for 95 (21.3%) instances of violence, patient family or relatives for 322 (72.4%), police or military personnel for 19 (4.3%), and other sources for 9 (2%). The proportion of violent events reported did not differ between male and female doctors, although characteristics varied. There were 415 of the 505 doctors who reported that violence had increased since the beginning of the pandemic, and many felt the situation would only get worse. COVID-19 has heightened tensions in an already violent health workplace, further increasing risks to patients and health providers.

**Interpretation:**

During the COVID-19 epidemic in Iraq an already violent hospital environment in Baghdad has only worsened. The physical and emotional toll on health workers is high which further threatens patient care and hospital productivity. While more security measures can be taken, reducing health workplace violence requires other measures such as improved communication, and addressing issues of patient care.

## Introduction

Violence against health care workers is defined as incidents where workers are abused, threatened or assaulted in relation to their work and involving an explicit or implicit challenge to their safety, well-being or health [1]. In some countries, workplace violence makes healthcare the most dangerous of occupations [2, 3]. In addition to the common verbal aggression and harassment, perhaps a third of health workers will be exposed to physical violence such as rape, injuries, kidnapping and death during their careers [4]. The true extent of violence against health worker is underreported, perhaps by half [5]. Increasingly the need to better document violence against health care has been cited, particularly during times of conflict or civil strife [6]. Perpetrators are commonly the family of patients, if not the patients themselves, but there may be tribal actions, and even popular or collective demonstrations against health-care workers. This is especially true in disaster and conflict situations. The consequences of violence to health care workers are substantial. Depression, anxiety, sleeplessness, and mental exhaustion are frequently reported [7]. Long term impacts include absenteeism, low job performance, burn-out, immigration, and leaving their profession. An atmosphere of health workplace violence has a negative effect on the provision of care [2].

The frequency of health workplace violence has increased in many places with the onset of the COVID-19 pandemic [8]. In addition to verbal and physical threats, there have been arson of testing facilities, targeting of health workers outside of facilities and violent responses to masking or physical distancing requirements. The widespread disinformation and politicization associated with the pandemic has most certainly increased the hostile environment against hospital as well as public health workers. This situation is further aggravated by the impact of the pandemic in conflict-affected countries. Insecurity Insight, a non-governmental organization, has mapped 1143 health workplace violence events in 20 conflict-affected countries during 2020, 412 of these directly related to Covid-19 [9].

The COVID-19-pandemic arrived in Iraq shortly after an intense fight to drive ISIS from Mosul, and at a time of civic protests against government corruption and its inability to provide basic services [10, 11]. The pandemic escalated rapidly in July 2020, filling hospitals and resulting in many deaths including health workers [12]. Since then new cases tapered off, only to spike again in early 2021. By June 14, 2021, some 1,254,643 cases were reported in Iraq (population 41 million), the largest number of cases in the Arab world [13].

Violence against health care workers in Iraq was a problem before COVID-19. Among 401 doctors displaced to Jordan, a 2010 study found 61% had fled Iraq after being subject of a violent event, and 75% of their households had experienced targeted violence [14]. Deaths among doctors in Iraq from intentional violence had reached 47.6/1000 doctors/year [15]. A 2018 study found 85% of health workers surveyed in Baghdad hospitals and Primary Health Care centers had been exposed to violence from patients or patient relatives [16]. The most common reasons stated was a perception that the quality of health care being provided was poor, and that medical supplies were insufficient. A medical student survey in 2018 noted that relationships between patients and hospital staff in hospitals was one of mutual mistrust [17].

To understand the pattern of health workplace violence after the arrival of COVID-19 in Iraq and its impact on doctors, we carried out this study among 505 doctors in 11 Baghdad hospitals working in direct patient care.

## Methods

A quantitative survey was conducted at 11 general hospitals in Baghdad in March and April 2021. Each general hospital had a designated COVID-19 ward and was specified as a COVID-19 treatment center by the Ministry of Health based on location, catchment area and resources available. Doctors from all departments at these hospitals were invited to participate with the assurance that no personal identifiers would be asked, and all answers would be anonymous and kept confidential. For those providing oral consent, self-administered paper questionnaires were provided directly to the doctors to be completed in the presence of the survey team, in case any questions arose. Questionnaires were collected by the survey team upon their completion. The selected hospitals were visited several times to ensure all doctors had an opportunity to participate.

The questions were developed based on experiences of Iraqi medical staff with hospital violence, perceptions of patient tensions, and their own personal stresses. Questions were linked with previous work examining risks of violence to health worker in Baghdad health facilities [15]. After testing the questionnaire, it was revised and used for training of the survey team prior to data collection. Forms requested no personal or privileged information and contained no unique identifiers. The survey team were graduate students in Community Medicine and experienced in conducting surveys. Data were entered into SPSS and analyzed in Baghdad and Baltimore using STATA (StataCorp, College Station, TX).

Descriptive analyses were conducted to understand health care worker experiences with violence. To explore differences in perceptions and experiences between subpopulations, Pearson’s chi-square and Fischer’s exact tests were conducted; *p*<0.05 were considered statistically significant.

The study was approved by the Scientific and Ethical Committee of the Department of Community Medicine, Al Mustansiriya University as 87-02-03-2021. The Institutional Review Board of the Johns Hopkins Bloomberg School of Public Health determined the data analysis not to be human subject research (IRB designation #15933).

## Results

Questionnaires were completed by 505 doctors, and participation declined by 10 who cited pressing clinical responsibilities. Because of ongoing clinical rotation of doctors, it was hard to judge the representativeness of the sample, but we estimate that more than 50% of doctors providing direct clinical care completed questionnaires. The demographic characteristics of the sample are set out in Table 1. Younger age groups predominated, with 222 (44%) being under age 30, and 310 (61.4%) female.

**Table 1:** Participant demographics.

		n (%)
Age (years)	<30	222 (44.0)
	30–39	188 (37.2)
	40–49	70 (13.9)
	≥50	25 (5.0)
Sex	Male	195 (38.6)
	Female	310 (61.4)

Of the 505 doctors there were 190 specialists, with the majority being family medicine (67), internal medicine (45), or general surgery (20). The remaining 315 were junior doctors who had not completed specialty training. During the previous six months, 360 doctors had been part of a specific hospital unit, the balance were the junior doctors on rotating schedules. Of these 360, 204 (56.7%) had been attached to emergency medicine departments.

The personal experiences with violence are shown in Table 2. Of the 505 doctors, 444 (88.3%) doctors reported verbal or physical violence in the past six months. Most reports of hospital violence were reported among the younger doctors (*p* = 0.001). There was no difference in reports of violence between male and female doctors. Among those with no specialty qualifications, 292/316 (92.4%) reported experiencing workplace violence, the highest of any group. For the seven categories of specialists interviewed, only pediatricians reported that less than 70% had been subjected to workplace violence in the past six months.

**Table 2:** Personal experience with violence, n (%).

Doctor experienced hospital violence in the past 6 months (verbal/physical, insult, assault, threats)		Age			Sex			Total
		<40years	≥40years		Male	Female		
		n = 410	n = 95	*p*-value	n = 195	n = 310	*p*-value	n = 505
		374 (91.2)	72 (75.8)	0.001	172 (88.2)	274 (88.4)	0.95	446 (88.3)
Persisting or residual injuries experienced^a^		9 (2.5)	4 (5.6)	0.16	7 (4.1)	6 (2.3)	0.27	13 (3.0)
Source of Violence	Patient’s family/relatives	264 (70.8)	58 (80.6)	0.19	126 (73.3)	196 (71.8)	0.003	322 (72.4)
	Patient	86 (23.1)	9 (12.5)		31 (18.0)	65 (23.4)		95 (21.3)
	Police or military	15 (4.0)	4 (5.6)		14 (8.1)	5 (1.8)		19 (4.3)
	Other	8 (2.1)	1 (1.4)		1 (0.6)	8 (2.9)		9 (2.0)
Time of day violence experienced	Day	229 (61.6)	58 (80.6)	0.003	83 (48.3)	204 (75.0)	0.001	287 (64.6)
	Night	121 (32.5)	10 (13.9)		76 (44.2)	55 (20.2)		131 (29.5)
	Day/Night	22 (5.9)	4 (5.6)		13 (7.6)	13 (4.8)		26 (5.9)
What was your immediate response to violence?	Did nothing	114 (32.3)	31 (43.1)	0.026	44 (26.0)	101 (39.5)	0.001	145 (34.1)
	Called hospital security	132 (37.4)	17 (23.6)		51 (30.2)	98 (38.3)		149 (35.1)
	Fought back	75 (21.2)	20 (27.8)		64 (37.9)	31 (12.1)		95 (22.4)
	Asked colleagues for help	25 (7.1)	1 (1.4)		7 (4.1)	19 (7.4)		26 (6.1)
	Took time off work	2 (0.6)	1 (1.4)		1 (0.6)	2 (0.8)		3 (0.7)
	Other	5 (1.4)	2 (2.8)		2 (1.2)	5 (2.0)		7 (1.6)
Did you report the event?		111 (30.8)	19 (26.4)	0.45	59 (34.7)	71 (27.1)	0.092	130 (30.1)
If no, why?	Afraid of Patient Family/Relatives	47 (29.7)	6 (16.7)	0.078	17 (22.7)	36 (30.3)	0.003	53 (27.3)
	Felt Ashamed	16 (10.1)	9 (25.0)		18 (24.0)	7 (5.9)		25 (12.9)
	It wasn’t Important	88 (35.8)	17 (32.1)		37 (33.0)	68 (36.4)		105 (35.1)
	No benefit to reporting	95 (38.6)	21 (39.6)		40 (35.7)	76 (40.6)		116 (59.8)
What measures were taken in your health facility concerning this event?	Nothing	275 (76.2)	52 (72.2)	0.77	125 (74.0)	202 (76.5)	0.07	327 (75.5)
	Pursue legal action	44 (12.2)	10 (13.9)		28 (16.6)	26 (9.8)		54 (12.5)
	Improve safety measures	42 (11.6)	10 (13.9)		16 (9.5)	36 (13.6)		52 (12.0)

Note: Data are presented as n (%)

^a^ Includes fractures, dislocations, lacerations, contusions, facial injuries, residual psychological trauma

Among healthcare workers who experienced violence, 417 (93.9%) who indicated that violence came from the patients or family members. There were statistically significant differences between male and female doctors in the circumstances of hospital violence. Violence directed at doctors by police or military was reported more commonly by male than female doctors, (18.1% vs 1.8%) whereas the reverse was true for violence from the patients themselves (23.4% vs 18.0% *p*<0.001). Female doctors more commonly reported daytime violence (75.0% vs 48.3% *p*<0.001), though this may have represented scheduling patterns. In the face of violent events, male doctors were more likely to fight the assailants (37.9% vs 12.1%) and female doctors were more likely to take no action (39.5% vs 26.0%) *p*<0.001). Male doctors more commonly reported feeling shame after an attack than female doctors (24.0% vs. 5.9% *p*<0.001). Younger doctors were more likely to express fear as a reason for not reporting a violent event to hospital authorities (29.7% vs 16.7% p = 0.039). The majority of attacks were verbal assaults or superficial contact, though fractured bones, lacerations, dislocations, contusions and residual psychological trauma occurred. No female doctors reported being physically attacked though one in five male doctors were struck by patients or family members during incidents.

Over 82% of participants (415) perceived that hospital violence increased following the arrival of COVID-19 (Table 3). There were 266 (52.7%) who expected violence to continue increasing for the future and 200 (39.6%) who expected it to stay about the same. Many reasons were suggested for the increased violence, from perceived poor quality of hospital care to popular unrest in the country. Current tension levels among patients and their family members were judged to be high by 329 doctors (65.1%) compared with only 122 doctors (24.2%) thinking tensions were high before the pandemic. Among doctors, self-reported stress levels were substantial. Deaths among fellow health workers from COVID-19 and concerns about bringing the infection to the family at home were major concerns reported. Anxiety was reported by 215 (42.5%) of doctors and depression by 122 (28.0%). Fear was more commonly reported emotion among female doctors (18.6% vs 9.5%) while insomnia was more common among males (12.5% vs 4.9%). Changes in reported tension levels reported were not significantly different among doctors exposed to hospital violence compared with those who were not.

**Table 3:** Hospital violence.

*Levels of hospital violence*		n (%)	
Has violence against doctors increased since the onset of the COVID-19 pandemic?		Yes	415 (82.2)
		No	90 (17.8)
If answered YES, what are probable reasons? (more than one response possible)			
Hospital security fails to protect medical staff		288 (57.0)	
Lack of medicines and supplies		266 (52.7)	
Patients perceived poor medical and health services		220 (43.6)	
Overload of COVID-19 cases on the intensive care unit		185 (36.7)	
People panic from risk of death in hospitals		180 (35.7)	
Aggressive attitude due to general population unrest		178 (35.2)	
Insufficient beds for the increased numbers of patients		177 (35.0)	
Rapid exacerbation of the illness leading to an unexpected death		141 (27.9)	
Fear of quarantine		126 (23.8)	
Patient relatives fear becoming infected while visiting in the hospital		101 (20.0)	
How do you think the level of hospital violence will change in the immediate future?	Will improve	39 (7.7)	
	Will get worse	266 (52.7)	
	No change from present	200 (39.6)	
How is the tension level now among patients and patient relatives compared to before COVID-19?	**Low**	**Medium**	**High**
	n (%)	n (%)	n (%)
Tension level before COVID-19	61 (12.1)	322 (63.8)	122 (24.2)
Tension level now	24 (4.8)	152 (30.1)	329 (65.1)
Compared to the beginning of the Covid-19 outbreak, how do you think your personal tension levels have changed?	Lessened		197 (39.0)
	Stayed the same		76 (15.0)
	Worsened		134 (26.5%)
	Fluctuated		98 (19.4%)
*Self-reported causes of stress affecting doctors*	Low importance	Some importance	High importance
Lack of initial knowledge in managing cases of COVID -19	67 (13.4)	208 (41.7)	224 (44.9)
Exhaustion and change in sleep pattern	116 (23.0)	173 (34.3)	215 (42.7)
Facing great changes and difficult adapting	66 (13.1)	201 (39.9)	237 (47.0)
Fear of being held accountable for patients’ death	66 (13.1)	168 (33.3)	271 (53.7)
Panic created by social media	79 (15.6)	147 (29.1)	279 (55.2)
Fear of contracting COVID-19	41 (8.1)	162 (32.1)	302 (59.8)
Lack of personal protective equipment (PPE)	54 (10.7)	144 (28.6)	306 (60.7)
Increase mortality among health workers	28 (5.5)	159 (31.5)	318 (63.0)
Lack of effective treatment for COVID-19	31 (6.2)	143 (28.4)	330 (65.5)
Worry about infecting my family at home	5 (1.0)	58 (11.5)	442 (87.5)
Have you experienced any psychological symptoms which you think are attributed to exposure to this violence?		Anxiety	215 (42.5)
		Depression	122 (28.0)
		Fear	65 (14.9)
		Insomnia	34 (7.8)
Do you think that the fear of violence negatively reflects on your professional performance in the hospital?		Yes	290 (57.7)
		Somewhat	160 (31.7)
		No	54 (10.7)
Do you think your own family tensions increased because you are working with COVID-19 patients?		Yes	442 (87.5)
		No	63 (12.5)

Younger doctors were more likely than older doctors to believe that hospital violence would negatively affect their professional performance (61.1% vs 42.1%) *p* = 0.003. Female and male doctors were equally concerned about effects on professional performance (58.4% vs 56.2%).

Doctors were asked about violent hospital events befalling their colleagues, and almost all had witnessed these firsthand or heard of such events (Table 4). Kidnapping and deaths were more commonly reported by older doctors. Violent events (all types) occurring to colleagues were reported more commonly by the younger doctors (*p* = 0.025). Some 453 (89.7%) respondents were aware of doctors who had emigrated specifically because of hospital violence.

**Table 4:** Impact of violence on health services reported by 505 doctors.

			n (%)
Have you witnessed/heard of any act of violence against other doctors?	Yes		490 (98.0)
	No		10 (2.0)
Have your colleagues been exposed to any form of violence?	Injured		349 (70.8)
	Kidnapped	19 (3.9)
	Killed	26 (5.3)
	Other forms*	99 (20.0)
Have you heard about any of colleagues who left Iraq specifically to avoid hospital violence?	Yes		453 (89.7)
	No		52 (10.3)
Do you feel local authorities are supporting you against violence?	No	Somewhat	Yes
	259 (51.4)	180 (35.7)	65 (12.9)
Do you feel safe from physical violence in your hospital unit?	335 (66.3)	121 (24.0)	49 (9.7)
Has your health facility increased security activities to protect staff against assaults since the COVID-19 pandemic began?	318 (63.3)	127 (25.3)	57 (11.4)
If yes; do you think this has reduced risk to hospital staff?	22 (21.7)	44 (41.5)	40 (37.7)
*Suggestions to control or limit the danger to health workers from patients and their families by categories*.			
Improve security			83 (16.4%)
Establish laws to protect health workers			75 (14.9%)
Punish and/or penalize the offender			35 (6.9%)
Educate and raise awareness within communities			30 (5.9%)
Improve service delivery and support health system			29 (5.7%)
Policy to reduce number of people accompanying patients			23 (4.6%)
HCWs leave the country (6) or workplace (1)			7 (1.4%)
Provide HCWs with a means for self-protection			5 (1.0%)
Other			(3.0%)

* Other: Shoved, kicked, threats, tribal penalties, verbal assaults

Most doctors felt there was little assistance to be expected from local authorities to reduce hospital violence. There were 335 (66.3%) who felt unsafe from violence in their hospital unit, although older doctors were more likely to feel more secure (17.9% vs 7.8% *p*<0.001). Despite the perceived increase in hospital violence with the pandemic, most doctors felt that there had been little or no improvements in hospital security. Among those who reported increased security efforts (11.4%), there were mixed opinions as to whether increased security measures had made the hospital any safer. Suggestions to lessen health worker violence centered on improved hospital security, taking legal and penal actions, and limiting numbers of patient visitors. A smaller number suggested improving underlying hospital care problems such as poor service quality. A few doctors said just accept it, as it was unlikely to change.

## Discussion

This study reveals a distressing pattern of increasing violence against Iraqi doctors during the COVID-19 pandemic amidst ongoing non-international armed conflict and popular demonstrations against corruption and dysfunctional governance. Reduced health resources and restricted salary related to low international oil prices and the economic impact of COVID-19 further complicated health care [18]. Within in the past six months nearly nine out of 10 doctors surveyed in the 11 Baghdad hospitals reported a verbal or physical assault. By a large majority, health workers see violence as substantially increased over an already prevalent pattern of violence in Iraqi hospitals [15]. This was vividly illustrated by a widely viewed video of Dr. Tariq Al-Sheibani being beaten unconscious by relatives of a patient dying from COVID-19 in Najaf [19]. In this current Baghdad study, the fear of being held accountable for a patient’s death was a common concern of doctors.

Although the majority of participating doctors in the 11 hospitals were female, violence was directed equally against male and female doctors, unlike reports from India of higher rates against female doctors [20]. However, physical assaults were directed only against male doctors, respecting Iraqi culture norms. Female doctors were more likely to report fear while working in the hospital setting, whereas male doctors were more likely to report insomnia, a characteristic observed as well among Chinese doctors exposed to hospital violence [6]. Depression and anxiety were common experiences noted by Iraqi doctors. The self-identified sources of stress were most commonly focused on personal risks and possibilities of bringing home infections to their families, the latter a common finding in health care settings during the pandemic [21]. While the fear of diminished professional performance in a violent hospital environment was common among Baghdad doctors, it was mostly expressed by the younger doctors, findings reported among health workers elsewhere [22]. Physical exhaustion, psychological stress, increased absenteeism, and fear of personal safety all can contribute to the diminished quality of care. It was also the younger doctors who were more likely to feel unsafe in the hospital setting. The frequent reporting of violent events occurring to the colleagues of the doctors participating suggests that violence was pervasive in Iraqi hospitals. Commonly these events were not reported to hospital authorities or hospital security, suggesting that doctors felt reporting would not lead to any change in hospital violence or feared repercussions from reporting.

The impact of violence on the mental health of health care workers only adds to the stress of providing pandemic care in difficult and under-resourced circumstances [8].{8} Anxiety, fear, exhaustion, depression, absenteeism, stigmatization, and doubting one’s professional skills are widely noted in these reports. Many Baghdad doctors dreaded reporting for duty each day. In the context of civil unrest, these stresses are likely to be increased, as noted by the Iraqi doctors. Internal displacement was noted as a factor in Libya, and is likely true in Iraq, though displacement was not specifically queried in our study [23].

While health workplace violence is a worldwide problem, its nature is shaped by local context. In some places, health workers are accused of spreading the diseases [9]. In the first half of 2020 some 365 COVID-19 linked attacks on health workers in 61 countries had occurred [24]. India and Mexico have been identified as hotspots of violence against health workers during the pandemic [25]. There have been several reports in India of community members attacking health workers and the families of doctors ostracized [26]. Fear, rumors and mistrust have suggested that health care workers are becoming the “newer untouchables” [27]. Difficulties with physician-patient communications has been identified as a weak point in reducing health worker violence in India. Mexico has seen a large number of attacks against health workers, many fueled by the misinformation about COVID-19, a widely reported problem in Latin America [28, 29]. A history of societal violence and mistrust of government have been cited as a predisposing factor in increased health care violence during the pandemic in Colombia, India and Libya [23].

Although there has been extensive documentation of the events and the reactions among health workers, less attention has been paid to the structural issues contributing to the violence. In this Baghdad study, the participating doctors categorized a number of contributing hospital or health systems issues. These largely paralleled pre-COVID-19 findings in surveys of Baghdad hospitals and PHC clinics as well as the opinions of medical students about relationships between health workers and patients [16, 17]. Patients and their families’ perceptions of poor health services, lack of medicine, hospital overload, and risks of family members becoming infected were commonly noted by the doctors. Many of these same factors were associated with health worker violence in Pakistan [30]. In India dissatisfaction with inefficient service systems, long waiting time, overcrowding, and few staff or resources, were thought to instigate episodes of violence [31]. Poor communication between doctors and patients was felt to be a major contributor in India. Inequities in access to services is a structural factor also identified in India [31]. In Iraq, insufficient hospital security measures were at the top of the list of reasons for violence in this study, and also noted previously in Baghdad hospitals [16].

Violent workforce events are widely underreported to hospital authorities in this study as well as elsewhere [5]. In our study underreporting was likely because doctors had limited expectations these incidents could be addressed with constructive approaches. Most respondents stated that the increased workplace violence during the pandemic had not caused hospitals to increase security, nor did they expect local authorities to be sympathetic. Among those doctors who reported that their hospital had increased security there was a perception that these measures had, to at least some extent, reduced the risk of hospital violence. Little study has been given to the characteristics of the perpetrators of violence. Some, such as disruptive behavior among adolescent males, alcohol intoxication, low educational attainment and persons accompanying emergency cases were more likely to be responsible for violence in an India study [31]. An alert hospital security service could identify such persons early, lessening the potential for violence and ensure some accountability for belligerent actions. A proactive institutional support for provider protection in Thailand proposed a multi-phased approach beginning with zero-tolerance policies and providing the skills to enforce them. A second phase includes a personal protection committee, a surveillance process, and aggressive behavior training programs for health workers [32].

There are measures that hospitals can take beyond security enforcement. Developing systematic, participatory, and culture-sensitive, non-discriminatory approaches for staff facing violence has been used [31]. Often these interventions are based on de-escalation methods, simulation methods, the sensitivity training for professionals, and changes in the health care management process. Training can help identify the warning signs of violent behavior to guide the development of prevention strategies to diffuse a violent situation. The need for hospitals and physicians to improve listening and communication skills has been identified [31]. Training can also help health workers with coping strategies when facing acts of violence and managing their longer-term emotions. Changes to the setting such as altering patient loads, restricting the number and types of visitors allowed, and the adjusting the size of health units could also be considered. Addressing the underlying dissatisfactions such as waiting times, access to services and medicines, staffing levels, and other quality of care matters are fundamental health systems issues with which Iraq has been struggling for many years.

Laws and ordinances have been frequently suggested as approaches to control workplace violence, and indeed this was the second most common suggestions from Baghdad doctors after that of improving security. Although legal statues have been reinforced in India to reduce hospital violence, their impact is still uncertain [33]. Finally, there were some Iraqi doctors who said the only option was to leave, either leaving the current hospital posting, or leaving the country.

A study of this nature has many limitations. Although the survey team made several visits to each hospital, some doctors were not included. We included hospitals designated for care of COVID-19, but hospitals without a COVID-19 ward may have shown a different pattern. Matching hospitals with COVID-19 wards with other Baghdad hospitals was not possible because of unique characteristics of designated hospitals with COVID-19 wards. The study did not include nurses or other health workers who may have had different exposures to hospital violence. We did not sample patient or patient families to understand their perceptions. Experience of Iraqi doctors dealing with the pandemic in other Iraqi cities may have been different. Questionnaires may not have captured many of the complexity of hospital violence or the complicated emotions generated in a complex and insecure environment. Finally, doctors may have been reticent to communicate sensitive events. In depth interviews about the origins of the violence, and interviews with patients, hospital management, and security personnel could have provided additional perspectives. This would be an important next step.

## Conclusions

The pandemic has exacerbated what was already a major problem with patient dissatisfaction and workplace violence in Baghdad hospitals. The extent of violence is most certainly underreported to hospital authorities. Younger doctors were especially affected by hospital violence. Male doctors were more likely to have been struck during violent incidents than were female doctors. The emotional impact of the current hospital status is great, with many doctors dreading reporting for work each day. The survey team noted that the doctors participating looked physically and mentally tired. Although some doctors reported increased hospital security, it was not clear how effective these increased efforts had been in reducing hospital violence toward doctors. Much of the patient dissatisfaction, which likely gives rise to the violence, is related to perceptions of the quality and adequacy of hospital care by patients and family members.
